# Survival benefit after radiotherapy for patients with malignant pleural mesothelioma: A propensity score‐matched study

**DOI:** 10.1002/mco2.241

**Published:** 2023-03-29

**Authors:** Liyou Lian, Huijun Lei, Shuwen Cheng, Rujie Zheng, Hongxia Yao, Jinfei Chen, Tianhui Chen

**Affiliations:** ^1^ Department of Oncology the First Affiliated Hospital of Wenzhou Medical University Wenzhou China; ^2^ Department of Cancer Prevention Zhejiang Cancer Hospital Hangzhou China; ^3^ Institute of Basic Medicine and Cancer (IBMC) Chinese Academy of Sciences Hangzhou China; ^4^ Department of Oncology Nanjing University of Medical School Nanjing China

Dear Editor,

Malignant pleural mesothelioma (MPM) has a poor prognosis, with a median survival time of less than 1 year once diagnosed. There are no curable methods for MPM, and most patients are prone to developing local progression and organ metastases.^1^ Consequently, almost all treatments are experimental or palliative, but not curable. So, it is still unclear what is the optimal treatment for mesothelioma. Even though there have been changes in care procedures throughout the years, patients’ clinical prognoses have improved only a little bit. Radiotherapy is one of the effective methods in the treatment of malignant tumors, but few studies are elucidated whether radiotherapy can be effective for MPM patients with or without distant metastases.

Our study aimed to evaluate whether radiotherapy could prolong the overall survival (OS) in MPM patients with or without specific organ metastases using the Surveillance, Epidemiology, and End Results (SEER) database from January 1, 2004, to December 31, 2015. The SEER*Stat software was used to identify MPM patients (Figure S1). We selected the pleural as the primary disease site and used the ICD‐O‐3 code (C38.4). Only patients diagnosed with ICD‐O‐3 histology/behavior codes of 9050/3‐9055/3 were included. Patients without the pathological diagnosis of MPM were excluded. The following parameters were collected from the sample: (a) age, (b) gender, (c) race, (d) marital status, (e) primary site of the tumor, (f) T stage, (g) N stage, (h) receipt of chemotherapy, (i) receipt of surgery, (j) receipt of radiation, (k) OS in months, and (l) organ metastases.

Overall, 7,299 MPM patients with pathological confirmation were extracted from the database (Table S1). The median age at diagnosis for the patients was 74 years old (interquartile range 18–100 years old), and 79% of the patients were male. A total of 803 (11%) patients underwent radiotherapy for the primary tumor. We performed 1:1 propensity score matching (PSM) between radiotherapy and no‐radiotherapy groups. After PSM, there were 803 patients in each group, and both groups had an average propensity score of 0.2 ± 0.01 (Figure S2). The baseline characteristics of radiotherapy and no‐radiotherapy groups were balanced after matching.

Before PSM, the median OS time of the radiotherapy group was longer than the no‐radiotherapy group (12 months vs. 8 months, respectively; hazards ratio [HR]: 0.761, 95% confidence interval [CI] 0.705–0.821, *p* < 0.001, Figure 1A). A total of 93 patients were complicated with bone metastases among the 7299 patients (except for lung, liver, and brain metastases). There was a statistical OS difference in MPM patients with bone metastases between the radiotherapy group and no‐radiotherapy group (6 months vs. 3 months, respectively; HR: 0.725, 95% CI 0.463–1.135, *p* = 0.16, Figure 1B). Besides, there were 142 MPM patients with lung metastases but without liver, bone, and brain metastases. Similarly, we performed KM analysis in lung metastases patients and found radiotherapy could reduce the OS (2 months vs. 8 months, Figure 1C). After balancing the baseline characteristics, we found MPM patients could still benefit from radiotherapy (HR: 0.868, 95% CI: 0.785–0.961, *Pp* = 0.006, Figure 1D,H). Additionally, patients treated with radiotherapy and surgery had longer OS than those treated with radiotherapy alone (19 months vs. 13 months, respectively; HR: 0.758, 95 CI%: 0.659–0.872, *p* < 0.001, Figure 1E,H). However, the OS of patients treated with radiotherapy and chemotherapy was no longer than those who were treated with chemotherapy alone (13 months vs. 12 months, respectively; HR: 0.970; 95% CI: 0.850–1.106, *p* = 0.644; Figure 1F,H). We also found that trimodality (combination with surgery, chemotherapy, and radiotherapy) could significantly prolong the OS of MPM patients, compared to radiotherapy with chemotherapy or surgery (20 months vs. 11 months; HR: 0.612, 95% CI: 0.518–0.722, *p* < 0.001, Figure 1G). We found that younger age, female, early TNM stage, surgery, chemotherapy, and radiotherapy were potential protective factors in MPM patients using univariate and multivariate regression analysis (Table S2). Radiotherapy was associated with better clinical outcomes in MPM patients before and after balancing the baseline characteristics. And MPM patients with bone or lung metastases could not benefit from radiotherapy.

**FIGURE 1:**
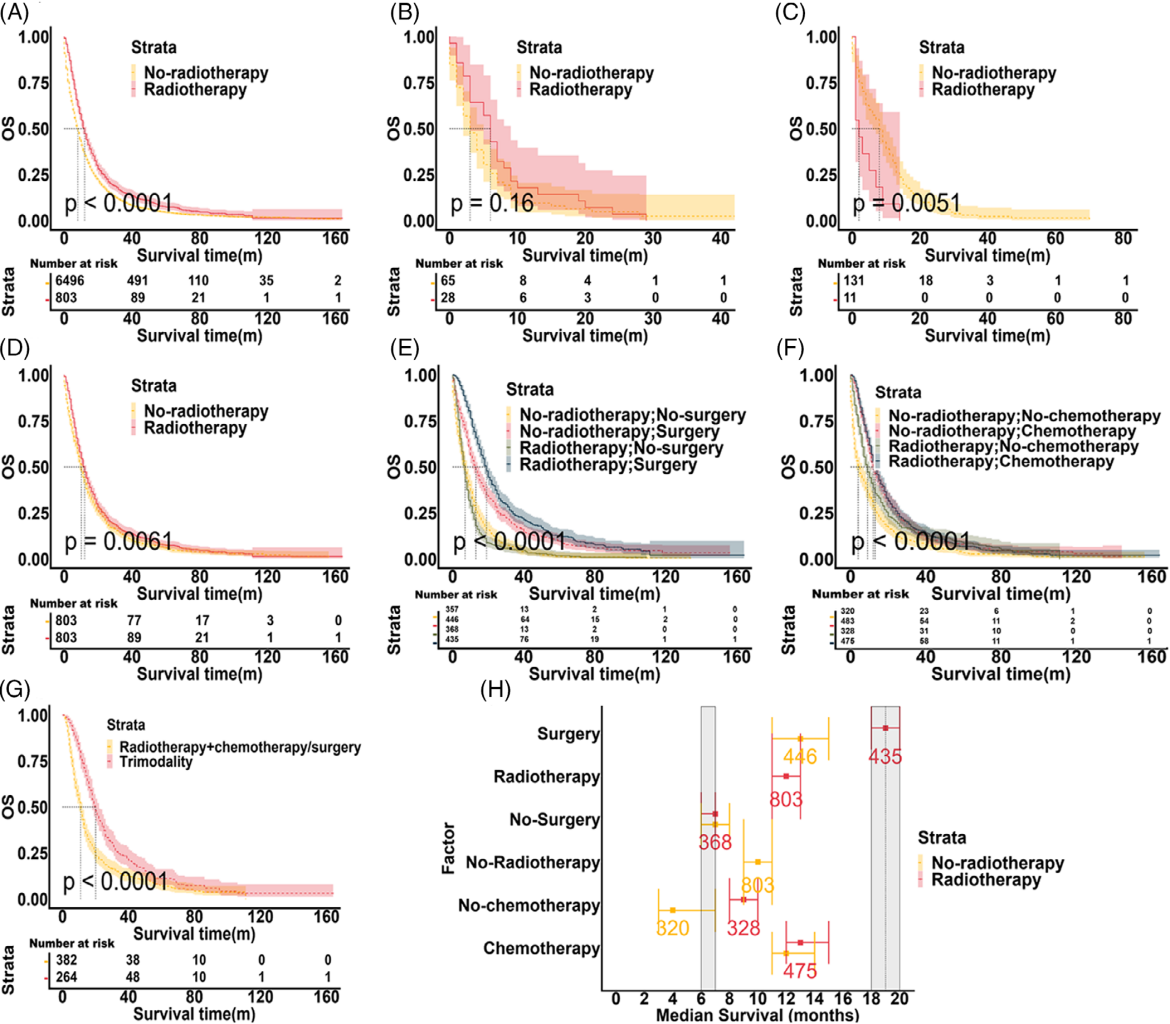
Clinical prognosis of malignant pleural mesothelioma (MPM) patients before and after propensity score matching. Kaplan‐Meier survival probability estimates and 95% confidence intervals for patients in no‐radiotherapy and radiotherapy groups before propensity score matching: (A) in the total sample, (B) in bone metastases, (C) in lung metastases, and after propensity score matching: (D) in the total sample, (E) receipt of surgery, (F) receipt of chemotherapy, (G) receipt of trimodality. (H) Median overall survival (months) ± 95% confidence intervals in MPM patients between radiotherapy and no‐radiotherapy groups.

According to an analysis based on the National Cancer Database, radiotherapy could prolong the OS among those with pathologic stage I‐II MPM, but not those who with pathologic stage III‐IV MPM.^2^ A phase II study showed that radiotherapy was effective in resectable MPM patients, which suggested that patients with extrapleural pneumonectomy after radiotherapy could have a better clinical prognosis.^3^ These findings were similar to our research, which demonstrated that radiotherapy was efficacious in MPM patients. Moreover, the results of our study displayed that radiotherapy was of no benefit to patients with bone or lung metastases. We speculated that the following factors might contribute to the results. First, MPM was highly malignant and had a worse prognosis. Once bone metastasized, the patient's OS would drastically reduce. Second, radiotherapy itself could cause several kinds of side effects and could cause various associated disorders. Once MPM was complicated with metastasizing, patients might not be able to withstand the pain of radiation and had poor outcomes. Finally, the number of lung or bone metastases in radiotherapy was small, which might influence the statistical power.

Our study also found that those patients treated with trimodality therapy, in combination with surgery, chemotherapy, and radiotherapy, had better clinical outcomes. An RCT showed that trimodality therapy, including radiotherapy, could improve the OS.^4^ A SEER database‐based analysis concluded that radiotherapy, as a component of trimodality therapy for patients with localized MPM, could significantly improve the OS.^5^ These findings supported significant benefits in OS that may be achieved by including radiotherapy as a component of trimodality therapy for patients with MPM, compared to solely employing chemotherapy and surgery.

We concluded that radiotherapy could improve the clinical prognosis of MPM patients. No survival advantage was observed among those who were complicated with bone or lung metastases. Although our study has several limitations (lack of specific radiation information and selection bias), the findings provide important clues supporting the use of radiotherapy in the MPM group. Further well‐designed clinical trials to investigate the effectiveness of radiotherapy in MPM were needed.

## AUTHOR CONTRIBUTIONS

All authors made a significant contribution to the work reported. Tianhui Chen and Jnfei Chen contributed to the conception, study design of the study; Liyou Lian took part in drafting the article; Shuwen Cheng and Huijun Lei took part in the acquisition of data, analysis, and interpretation; Rujie Zheng and Hongxia Yao took part in collecting data. All also contributed to revising or critically reviewing the article. All authors have read and approved the final manuscript.

## CONFLICT OF INTEREST STATEMENT

All authors declare no conflict of interest.

## FUNDING INFORMATION

This work was supported by the National Key Research‐Development Program of China (2019YFE0198800), Ten‐Thousand Talents Plan of Zhejiang Province (2021R52020) and The First Affiliated Hospital of Wenzhou Medical University (2021QD025).

## ETHICS STATEMENT AND INFORMED CONSENT

SEER data were completely anonymous and their use did not entail ethical problems.

## Supporting information

Supporting Information

## Data Availability

Primary data from the occupational screenings are available upon reasonable request. The datasets generated for the claims data cohort are not available as the use of claims data is restricted to defined persons
