# Supplementary material for: Survival benefit after radiotherapy for patients with malignant pleural mesothelioma: A propensity score‐matched study
Source: MedComm (2020). 2023 Mar 29;4(2):e241. doi: 10.1002/mco2.241 (PMC10060947; doi:10.1002/mco2.241)

**Title page**

**Title:**

Survival benefit after radiotherapy for patients with malignant pleural mesothelioma: A propensity score-matched study

**Running title:**

Survival after radiotherapy for MPM

**Author:**

Liyou Lian^1^, Huijun Lei^2^, Shuwen Cheng^3^, Rujie Zheng^1^, Hongxia Yao^1^, Jinfei Chen^1, *^, Tianhui Chen^2,*^

**Affiliations:**

^1^the First Affiliated Hospital of Wenzhou Medical University, Wenzhou 325000, the People's Republic of China;

^2^Department of Cancer Prevention/Zhejiang Cancer Institute, Cancer Hospital of the University of Chinese Academy of Sciences (Zhejiang Cancer Hospital); Institute of Basic Medicine and Cancer (IBMC), Chinese Academy of Sciences, Hangzhou 310022, China; Department of Preventive Medicine, School of Medicine, Ningbo University, Ningbo 315211, the People's Republic of China;

^3^Nanjing University of Medical School, Nanjing 210046, the People's Republic of China;

**^*^Correspondence Author:**

Prof. Tianhui Chen, Department of Cancer Prevention/Zhejiang Cancer Institute, Cancer Hospital of the University of Chinese Academy of Sciences (Zhejiang Cancer Hospital); Institute of Basic Medicine and Cancer (IBMC), Chinese Academy of Sciences, Hangzhou 310022, China; Department of Preventive Medicine, School of Medicine, Ningbo University, Ningbo 315211, the People's Republic of China; Email: chenth@zjcc.org.cn;

Prof. Jinfei Chen, the First Affiliated Hospital of Wenzhou Medical University, Wenzhou 325000, the People's Republic of China; E-mail: jinfeichen@sohu.com.

**Table S1** Baseline characteristics of enrolled patients

| **Variables** | **Before-PSM** | | **P-value** | **After-PSM** | **P-value** |
| --- | --- | --- | --- | --- | --- |
|  | **No-radiotherapy (N=6496)** | **Radiotherapy (N=803)** |  | **No-radiotherapy (N=803)** |  |
| **Age** |  |  | < 0.001 |  | 0.298 |
| Mean (SD) | 73.0 (10.8) | 67.8 (10.9) |  | 68.4 (11.2) |  |
| Median (Min, Max) | 74.0 (18.0, 100) | 68.0 (27.0, 93.0) |  | 69.0 (22.0, 97.0) |  |
| **Gender** |  |  | 0.999 |  | 0.578 |
| Female | 1368 (21.1%) | 169 (21.0%) |  | 159 (19.8%) |  |
| Male | 5128 (78.9%) | 634 (79.0%) |  | 644 (80.2%) |  |
| **Race** |  |  | 0.079 |  | 0.991 |
| Black | 336 (5.2%) | 27 (3.4%) |  | 26 (3.2%) |  |
| Other | 232 (3.6%) | 22 (2.7%) |  | 24 (3.0%) |  |
| Unknown | 11 (0.2%) | 1 (0.1%) |  | 1 (0.1%) |  |
| White | 5917 (91.1%) | 753 (93.8%) |  | 752 (93.6%) |  |
| **Marital status** |  |  | 0.135 |  | 0.384 |
| Married | 5164 (79.5%) | 661 (82.3%) |  | 681 (84.8%) |  |
| Single | 1083 (16.7%) | 119 (14.8%) |  | 104 (13.0%) |  |
| Unknown | 249 (3.8%) | 23 (2.9%) |  | 18 (2.2%) |  |
| **T** |  |  | < 0.001 |  | 0.414 |
| T0-T2 | 3480 (53.6%) | 281 (35.0%) |  | 284 (35.4%) |  |
| T3-4 | 2197 (33.8%) | 464 (57.8%) |  | 474 (59.0%) |  |
| TX | 819 (12.6%) | 58 (7.2%) |  | 45 (5.6%) |  |
| **N** |  |  | < 0.001 |  | 0.144 |
| N0-N1 | 4643 (71.5%) | 597 (74.3%) |  | 618 (77.0%) |  |
| N2-N3 | 700 (10.8%) | 124 (15.4%) |  | 97 (12.1%) |  |
| NX | 1153 (17.7%) | 82 (10.2%) |  | 88 (11.0%) |  |
| **M** |  |  | < 0.001 |  | 0.833 |
| M0 | 4674 (72.0%) | 598 (74.5%) |  | 606 (75.5%) |  |
| M1 | 1160 (17.9%) | 173 (21.5%) |  | 169 (21.0%) |  |
| MX | 662 (10.2%) | 32 (4.0%) |  | 28 (3.5%) |  |
| **Surgery** |  |  | < 0.001 |  | 0.616 |
| No | 5054 (77.8%) | 368 (45.8%) |  | 320 (39.9%) |  |
| Yes | 1442 (22.2%) | 435 (54.2%) |  | 483 (60.1%) |  |
| **Chemotherapy** |  |  | < 0.001 |  | 0.722 |
| No | 3535 (54.4%) | 328 (40.8%) |  | 357 (44.5%) |  |
| Yes | 2961 (45.6%) | 475 (59.2%) |  | 446 (55.5%) |  |
| **Histologic** |  |  | < 0.001 |  | 0.894 |
| 9050 | 2827 (43.5%) | 240 (29.9%) |  | 241 (30.0%) |  |
| 9051 | 768 (11.8%) | 127 (15.8%) |  | 119 (14.8%) |  |
| 9052 | 2396 (36.9%) | 348 (43.3%) |  | 360 (44.8%) |  |
| 9053 | 505 (7.8%) | 88 (11.0%) |  | 83 (10.3%) |  |

9050, mesothelioma; 9051, fibrous mesothelioma; 9052, epithelioid mesothelioma; 9053, mesothelioma, biphasic; 9055, multicystic mesothelioma

Abbreviations: PSM, propensity score matching.

**Table S2** Univariate and multivariate regression models for potential risk factors

| **Variables** | **Univariate regression** | | **Multivariable regression** | |
| --- | --- | --- | --- | --- |
|  | **HR (95% CI)** | **P value** | **HR (95% CI)** | **P value** |
| **Age Category** |  |  |  |  |
| < 50 | 1* |  | 1 |  |
| 50-69 | 1.429 (1.121-1.846) | 0.004 | 1.415 (1.098-1.823) | 0.007 |
| ≥70 | 2.314(1.801-2.971) | < 0.001 | 1.868 (1.446-2.412) | < 0.001 |
| **Gender** |  |  |  |  |
| Female | 1 |  | 1 |  |
| Male | 1.451 (1.275-1.651) | < 0.001 | 1.351 (1.182-1.543) | < 0.001 |
| **Race** |  |  |  |  |
| White | 1 |  |  |  |
| Black | 1.154 (0.875-1.522) | 0.311 | - | - |
| Other | 1.114 (0.828-1.500) | 0.475 | - | - |
| Unknown | 0.372 (0.052-2.645) | 0.323 | - | - |
| **T stage** |  |  |  |  |
| T0-T2 | 1 |  | 1 |  |
| T3-4 | 1.180 (1.059-1.316) | 0.003 | 1.099 (0.983-1.228) | 0.096 |
| TX | 1.839 (1.485-2.278) | < 0.001 | 1.145 (0.900-1.457) | 0.270 |
| **N stage** |  |  |  |  |
| N0-N1 | 1 |  | 1 |  |
| N2-N3 | 1.265 (1.094-1.463) | 0.002 | 1.430 (1.234-1.658) | < 0.001 |
| NX | 1.701 (1.446-2.001) | < 0.001 | 1.395 (1.152-1.690) | 0.006 |
| **M stage** |  |  |  |  |
| M0 | 1 |  | 1 |  |
| M1 | 1.800 (1.591-2.037) | < 0.001 | 1.661 (1.457-1.893) | < 0.001 |
| MX | 1.503 (1.156-1.954) | 0.002 | 0.901 (0.666-1.219) | 0.499 |
| **Histology** |  |  |  |  |
| 9050 | 1 |  | 1 |  |
| 9051 | 1.562 (1.334-1.826) | < 0.001 | 1.562 (1.330-1.834) | < 0.001 |
| 9052 | 0.548 (0.486-0.618) | < 0.001 | 0.678 (0.597-0.770) | < 0.001 |
| 9053 | 0.837 (0.701-1.000) | 0.050 | 1.152 (0.957-1.388) | 0.135 |
| **Surgery** |  |  |  |  |
| No | 1 |  | 1 |  |
| Yes | 0.443 (0.399-0.492) | < 0.001 | 0.576 (0.513-0.647) | < 0.001 |
| **Chemotherapy** |  |  |  |  |
| No | 1 |  | 1 |  |
| Yes | 0.697 (0.629-0.773) | < 0.001 | 0.694 (0.624-0.773) | < 0.001 |
| **Radiotherapy** |  |  |  |  |
| No | 1 |  | 1 |  |
| Yes | 0.868 (0.785-0.961) | 0.006 | 0.874 (0.789-0.968) | 0.010 |

9050, mesothelioma; 9051, fibrous mesothelioma; 9052, epithelioid mesothelioma; 9053, mesothelioma, biphasic; 9055, multicystic mesothelioma

Abbreviations, HR, hazard ratio; CI, confidence interval

* Means the control group

**Figure S1** Flow chart depicting the MPM patient selection process. Abbreviations: MPM, malignant pleural mesothelioma; SEER, surveillance, epidemiology, and end results.


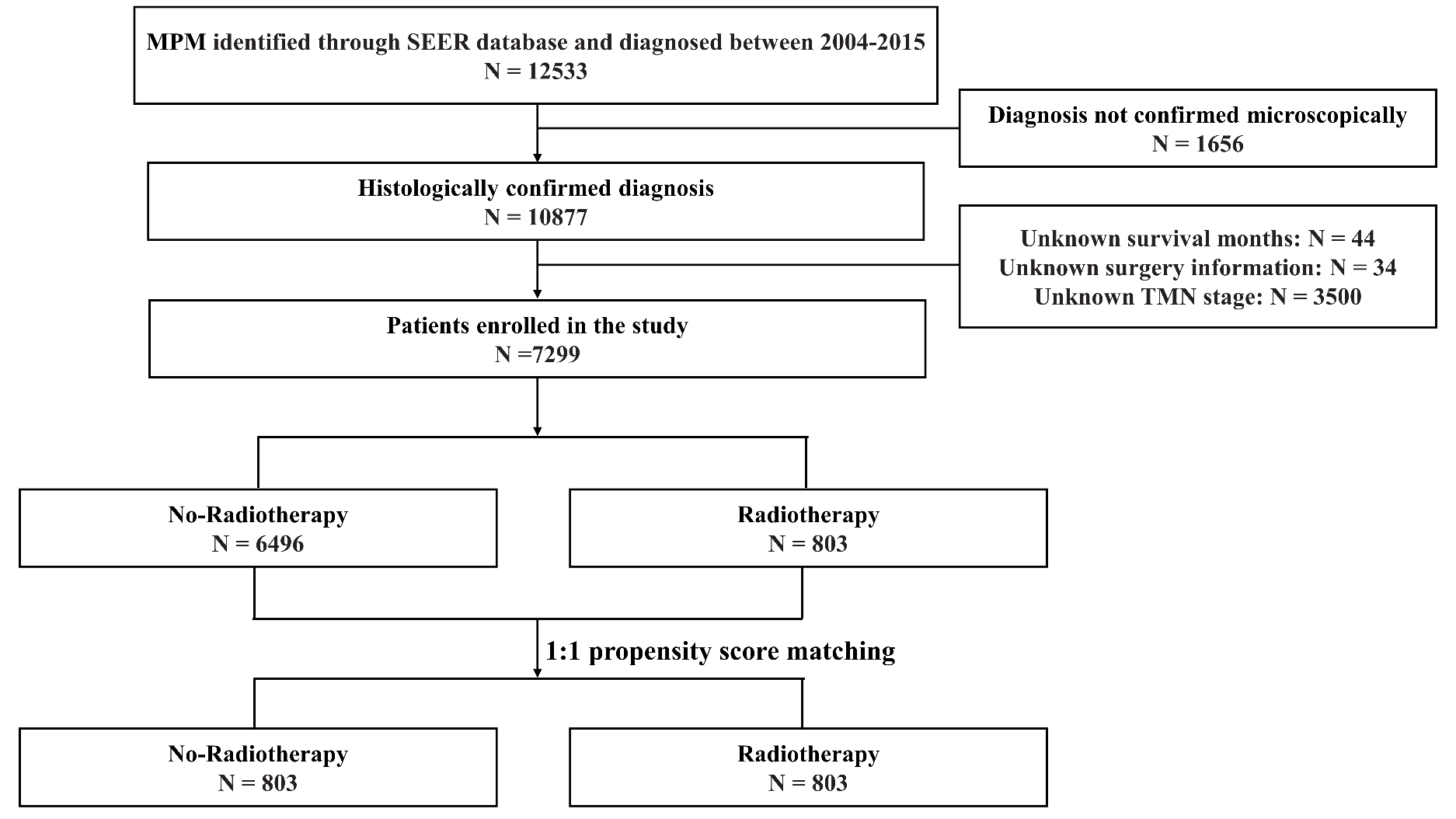


**Figure S2** Propensity score distribution between no-radiotherapy and radiotherapy groups in MPM patients. (A), (C) before and (B), (D) after propensity score matching. Abbreviations: MPM, malignant pleural mesothelioma.


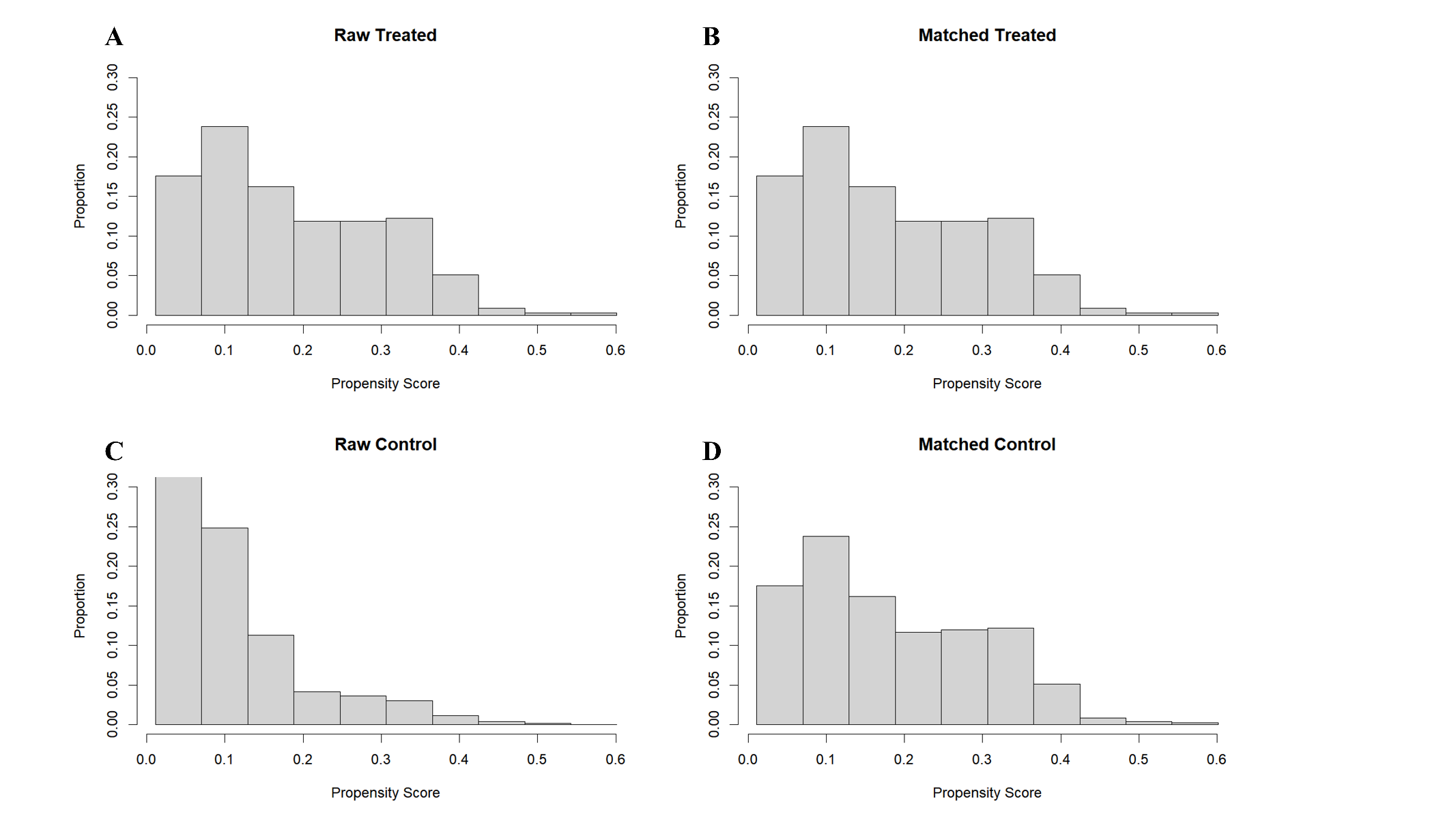

Supplement: Supplementary file 1 — Supporting Information [file MCO2-4-e241-s001.docx]
